# ﻿Two new species of *Scolecobasidium* (Venturiales, Sympoventuriaceae) associated with true mangrove plants and *S.terrestre* comb. nov.

**DOI:** 10.3897/mycokeys.96.100621

**Published:** 2023-03-29

**Authors:** Shuang Song, Meng Li, Jun-En Huang, Fang Liu

**Affiliations:** 1 State Key Laboratory of Mycology, Institute of Microbiology, Chinese Academy of Sciences, Beijing, 100101, China Institute of Microbiology, Chinese Academy of Sciences Beijing China; 2 University of Chinese Academy of Sciences, Beijing, 100049, China University of Chinese Academy of Sciences Beijing China

**Keywords:** Mangrove, phylogeny, plant pathogen, taxonomy

## Abstract

*Scolecobasidium* is cosmopolitan and includes species that inhabit a wide range of ecosystems including soil, water, air, plant and cold-blooded vertebrates. During a fungal survey from mangrove, strains of *Scolecobasidium* occurring on leaf spots of true mangrove plants, *Aegicerascorniculatum* and *Acanthusebracteatus*, were isolated from Futian Mangrove in Shenzhen and the Qi’ao-Dangan Island Mangrove in Zhuhai, China. Unlike most species in *Scolecobasidium* that produce dark conidia, our strains are characterized by hyaline to pale brown conidia and inconspicuous thread-like sterigmata. Further detailed morphological comparison and multi-locus (LSU, ITS, *tub2*, *tef1*-α) phylogenetic analyses revealed these collections as two new taxa, namely *S.acanthi***sp. nov.** and *S.aegiceratis***sp. nov.** We further emend the generic description of *Scolecobasidium*, propose one new combination, *S.terrestre* comb. nov., and clarify the taxonomic status of *S.constrictum*.

## ﻿Introduction

*Scolecobasidium* was described based on two species, *S.terreum* and *S.constrictum*, with the former as the generic type (Abbott 1927). This genus is slow-growing, and characterized by brownish to black colonies, reduced, hyaline or pigmented conidiophores and septate, smooth- or rough-walled, brown, single, dry and rhexolytic conidia (Abbott 1927; Barron and Busch 1962; Seifert and Gams 2011). A distinguishing feature of *Scolecobasidium* from other genera is the conidiophores, which are born on aerial hyphae as short non-septate structures producing conidia from the ends of thread-like sterigmata (Abbott 1927). Subsequently, more species with unbranched conidia were described in *Scolecobasidium* and the conidia of this genus are found to be variable in shape, especially its type species *S.terreum* producing Y-shaped or T-shaped conidia distinct from other species in the genus. Therefore, Hoog and von Arx (1973) introduced a separate genus *Ochroconis*, typified by *O.constricta* (syn. *S.constrictum*), and transferred many *Scolecobasidium* species into this genus (De Hoog 1973). At that time, *Ochroconis* was comprised of species with sympodial conidiogenesis and ellipsoidal, clavate or fusiform conidia, whereas the genus *Scolecobasidium* was restricted to species with T- or Y-shaped or bilobed, 1- or multiple septate conidia born on ampulliform conidiogenous cells possessing 1–3 conidial-containing denticles at the tip of the conidiophores (De Hoog 1973). Subsequently, Ellis (1976) classified *Ochroconis* as a synonym of *Scolecobasidium*, and Gams (2015) and Seifert and Gams (2011) agreed with this interpretation. In addition to the similar morphological characteristics of *Scolecobasidium* and *Ochroconis*, recent molecular analyses have clearly shown that the two genera constituted a polyphyletic complex and *Ochroconis* should not be treated as a separate genus (Hao et al. 2012; Samerpitak et al. 2014). Although the ex-type strains of both *S.terreum* (CBS 203.27) and *O.constricta* (CBS 202.27) are sterile after a long period of preservation, they were phylogenetically placed within the *Scolecobasidium* clade (Shen et al. 2020). Therefore, based on the principle of priority, the older generic name *Scolecobasidium* was chosen over *Ochroconis*, and 25 new combinations have been proposed (Shen et al. 2020; Crous et al. 2021; Wei et al. 2022).

*Scolecobasidium* is the largest genus within *Sympoventuriaceae*, *Venturiales*, *Dothideomycetes* (Shen et al. 2020) and about 98 epithets are currently listed in Index Fungorum (Index Fungorum accession date: 10.01.2023). *Scolecobasidium* is a cosmopolitan genus of saprotrophic soil hyphomycetes, some of which are also parasitic on plants (De Hoog 1985; Crous et al. 2016), human (Giraldo et al. 2014), fish (Ross and Yasutake 1973) or other animals (VanSteenhouse et al. 1988; Singh et al. 2006). In our study, during the fungal investigations of mangrove plants in China, several strains of *Scolecobasidium* were isolated from leaf spots on *Aegicerascorniculatum* and *Acanthusebracteatus*, and they were revealed as two novel species through polyphasic analyses. In addition, on the basis of Shen et al. (2020) and Wei et al. (2022), we correct the taxonomic status of ambiguous species in this group.

## ﻿Materials and methods

### ﻿Sample collection and fungal isolation

During our fungal investigations on mangrove plants in China, 90 strains of 48 species have been isolated from true mangrove plants, *Acanthusebracteatus* and *Aegicerascorniculatum* (Table 1). Among them, *Scolecobasidium*-like strains piqued our interest because their unique characters differed from known species and were further studied herein. Type specimens made from ex-type strains of the novel species were preserved in the Fungarium (**HMAS**), Institute of Microbiology, CAS, and the living cultures were preserved in the China General Microbiological Culture Collection Center (**CGMCC**) and LC culture collection (personal culture collection of Lei Cai housed in the Institute of Microbiology, Chinese Academy of Sciences).

**Table 1.** Pathogens and endophytes associated with true mangrove plant *Acanthusebracteatus* and *Aegicerascorniculatum*.

Strains No.	Fungus species	Host plant	Remark
SS1	*Acrocalymmamedicaginis*	*Ac.ebracteatus*	Pathogen
ds0003, SS2012, SS2094, SS2108, SS2009, SS2017, SS2096, SS2103	*Alternariaangustiovoidea*	*Ac.ebracteatus*	Endophyte
SS2047	*Arthriniumxenocordella*	*Ae.corniculatum*	Endophyte
ds0016	*Cercosporabeticola*	*Ae.corniculatum*	Endophyte
SS2097, SS27	*Cladosporiumaustrohemisphaericum*	*Ae.corniculatum*	Endophyte
ds1001, ds1030, SS10	*Cladosporiumcladosporioides*	*Ae.corniculatum*	Pathogen
SS2010	*Cladosporiumcolombiae*	*Ae.corniculatum*	Endophyte
ds0017, ds1043, ds1032, ds1042	*Cladosporiumdominicanum*	*Ae.corniculatum*	Endophyte
SS8, SS42	*Cladosporiumoryzae*	*Ac.ebracteatus*	Pathogen
SS2110	*Cladosporiumrugulovarians*	*Ae.corniculatum*	Endophyte
ds1038-2	*Cladosporiumsphaerospermum*	*Ae.corniculatum*	Pathogen
SS28	*Cladosporiumtenuissimum*	*Ac.ebracteatus*	Pathogen
ds0011	*Colletotrichumgigasporum* complex	*Ac.ebracteatus*	Endophyte
SS2109, SS2046	*Cytospora* sp. nov.	*Ae.corniculatum*	Endophyte
SS2107	*Diaporthehongkongensis*	*Ae.corniculatum*	Endophyte
SS2041	*Diaportheperseae*	*Ae.corniculatum*	Endophyte
ds1038, SS2106	*Fusariumincarnatum*	*Ae.corniculatum*	Pathogen
ds1018	*Fusariumluffae*	*Ae.corniculatum*	Pathogen
SS14	*Fusariumsolani*	*Ae.corniculatum*	Pathogen
ds1045	*Halorosellinia* sp. nov.	*Ae.corniculatum*	Pathogen
ds0021, ds0022	*Haloroselliniaxylocarpi*	*Ac.ebracteatus*	Endophyte
ds1093, ds1094, ds1095	*Hortaeawerneckii*	*Ae.corniculatum*	Pathogen
ds1044	*Hypocreales* sp. nov.	*Ae.corniculatum*	Pathogen
SS29	*Nemania* sp. nov.	*Ae.corniculatum*	Endophyte
ds1087	*Neodevriesiatabebuiae*	*Ae.corniculatum*	Pathogen
SS2015, SS2045, SS2100, ds1062, ds1075	*Neofusicoccumkwambonambiense*	*Ae.corniculatum*	Endophyte
ds1060	*Neopestalotiopsiseucalypticola*	*Ae.corniculatum*	Pathogen
ds1020	*Neopestalotiopsisphangngaensis*	*Ae.corniculatum*	Pathogen
ds1061	*Neopestalotiopsis* sp. nov.	*Ae.corniculatum*	Pathogen
SS2092	*Nigrosporaoryzae*	*Ae.corniculatum*	Endophyte
ds1025	*Occultifur* sp. nov.	*Ac.ebracteatus*	Pathogen
SS2069, SS2038, SS2067, SS2068	*Penicilliumbrevicompactum*	*Ac.ebracteatus*	Endophyte
SS2051, SS2048, SS2052, SS2056, SS2058, SS2066, SS2077, SS2081, SS2083	*Penicilliumchrysogenum*	*Ae.corniculatum*	Endophyte
SS2087	*Penicilliumcoffeae*	*Ae.corniculatum*	Endophyte
ds1019	*Pestalotiopsiskandelicola*	*Ae.corniculatum*	Pathogen
SS2011	*Phyllostictacapitalensis*	*Ac.ebracteatus*	Endophyte
ds1081	*Phyllosticta* sp. nov.	*Ae.corniculatum*	Pathogen
SS20	*Pseudopestalotiopsischinensis*	*Ae.corniculatum*	Pathogen
ds1028	*Rhodotorulasphaerocarpa*	*Ac.ebracteatus*	Pathogen
ds1031	*Roussoellamediterranea*	*Ae.corniculatum*	Pathogen
LC19368	*Scolecobasidiumacanthi* sp. nov.	*Ac.ebracteatus*	Pathogen
LC19369, LC19370	*Scolecobasidiumaegiceratis* sp. nov.	*Ae.corniculatum*	Pathogen
SS2089	*Stemphyliumsolani*	*Ae.corniculatum*	Endophyte
ds1100	*Symmetrosporamarina*	*Ae.corniculatum*	Pathogen
ds1084	*Thyridiumpluriloculosum*	*Ae.corniculatum*	Pathogen
SS2044	*Tricharinaochroleuca*	*Ac.ebracteatus*	Endophyte
SS2040, SS2043	*Trichodermaharzianum*	*Ae.corniculatum*	Endophyte
ds1086, ds1026, ds1027, ds1079, ds1082, ds1026-2, ds1037	*Zasmidiumanthuriicola*	*Ae.corniculatum*	Pathogen

### ﻿Morphological observations

Colony features including color and growth rate were recorded for the strains grown on oatmeal agar (OA) and malt extract agar (MEA) after 14 days at 25 °C. To enhance sporulation, strains were incubated at 25 °C under near UV light with a 12 h photoperiod for 14 d or longer period. Morphological observations of reproductive structures were made in lactic acid and observed using a Nikon Eclipse 80i microscope using differential interference contrast (DIC) illumination. At least 30 measurements per structure were taken, and the mean value, standard deviation, and minimum–maximum values were given.

### ﻿DNA extraction, PCR amplification and sequencing

Fresh fungal mycelia grown on potato dextrose agar (PDA) for 14 d at 25 °C were scraped from the colony margin and used for genomic DNA extraction using a modified CTAB protocol as described previously (Guo et al. 2000). Genomic DNA was diluted to 1 ng/μL using sterile water as the template for PCR. The amplification of internal transcribed spacer (ITS) region including the flanking 5.8S rRNA gene, was carried using the primer pairs ITS1 and ITS4 (White, Bruns, Lee, Taylor, 1990), the 28S nuclear large subunit (nuLSU) with LR0R/LR5 (White, Bruns, Lee, Taylor, 1990), with EF1-728F/EF-2 (Qiao et al. 2016) for the partial translation elongation factor 1-alpha gene (*tef1*-α) and Bt2a/Bt2b to amplify the partial beta-tubulin gene (*tub2*) (Glass and Donaldson 1995), respectively. The reaction volume of 25 μL consisted of 10× PCR buffer 2.5 μL, MgCl_2_ 2 mM, dNTPs 50 μm/L, forward and reverse primers 0.1 μm/L, DNA polymerase 0.5 U, and DNA template 10 ng. PCR amplification reactions for LSU and ITS were performed as follows: pre-denaturation at 95 °C for 10 min, followed by 35 cycles of denaturation at 95 °C for 45 s, annealing at 52 °C for 45 s, extension at 72 °C for 1 min, and a final extension step at 72 °C for 10 min, but the annealing temperature was adjusted to 56 °C for *tef1*-α and *tub2*. PCR products were detected by 1% agarose gel electrophoresis and then sequenced by SinoGenoMax. MEGA v. 7 was used to obtain consensus sequences from DNA data generated from forward and reverse primers.

### ﻿Phylogenetic analyses

Phylogenetic analysis was performed using sequences of LSU, ITS, *tub2*, and *tef1*-α from 64 type and reference strains of *Ochroconis*, *Scolecobasidium* and one outgroup *Verruconiscalidifluminalis*CBS 125818 (Table 2). Single locus alignment was performed using an online version of MAFFT v. 7 (Katoh and Standley 2013) and then concatenated for Maximum Likelihood (ML) and Bayesian analysis (BA). ML and BA were implemented using RAxML-HPC BlackBox v. 8.2.12 and MrBayes v. 3.2.7a, respectively, in the CIPRES Science Gateway portal (https://www.phylo.org/; Miller et al. 2010). For ML analysis, GTR+GAMMA substitution model with 1,000 bootstrap iterations was set. For BA, MrModeltest v. 2.4 (Guindon and Gascuel 2003; Matic et al. 2012) was firstly used to determine the best evolutionary model for each locus. Bayesian analysis was computed with four simultaneous Markov Chain Monte Carlo chains, 10,000,000 generations, and a sampling frequency of 1,000 generations, ending the run automatically when standard deviation of split frequencies fell below 0.01. The burn-in fraction was set to 0.25, after which the 50% majority rule consensus trees and posterior probability (PP) values were calculated. The resulting trees were plotted using FigTree v. 1.4.4 (http://tree.bio.ed.ac.uk/software/figtree) and the layout was edited in Adobe Illustrator 2020. Newly obtained sequences in this study are submitted to GenBank. New descriptions and nomenclature were deposited in MycoBank (www.MycoBank.org) (Crous et al. 2014).

**Table 2.** Strains used in the phylogenetic analysis of *Scolecobasidium* and GenBank accession numbers.

Species	Strain^a^	Genbank accession numbers^b^			
		ITS	LSU	*tub2*	*tef1*
***acanthi***	**CGMCC 3.24352 = LC19368^T^**	**OQ448957**	**OQ448949**	**OQ442218**	**OQ442215**
***S.aegiceratis***	**CGMCC 3.24353 = LC19369^T^**	**OQ448958**	**OQ448950**	**OQ442219**	**OQ442216**
***S.aegiceratis***	**CGMCC 3.24354 = LC1937**0	**OQ448959**	**OQ448951**	**OQ442220**	**OQ442217**
*S.ailanthi*	MFLU 18-2110	MK347731	‒	MK412881	‒
*S.ailanthi*	MFLUCC 17-0923^T^	MK347730	MK347947	MK412883	–
*S.anellii*	CBS 284.64^T^	FR832477	KF156138	KF156184	KF155995
*S.anomalum*	CBS 131816^T^	HE575201	KF156137	KF156194	KF155986
*S.aquaticum*	CBS 140316^T^	KX668258	KX668259	–	–
*S.bacilliforme*	CBS 100442^T^	KP798632	KP798635	KT272059	KT272070
*S.blechni*	CBS 146055^T^	MN562134	MN567641	MN556843	MN556826
*S.camellicola*	GUCC 18242^T^	MZ503728	MZ503761	MZ546907	MZ546874
*S.capsici*	CBS 142096^T^	KY173427	KY173518	–	–
*S.constrictum*	CBS 202.27^T^	MH854929	MH866423	KF156161	KF156003
*S.cordanae*	CBS 412.51	HQ667540	KF156123	KF156200	KF155980
*S.cordanae*	CBS 475.80^T^	KF156022	KF156122	KF156197	KF155981
*S.crassihumicola*	CBS 120700	KJ867429	KJ867430	KJ867433	KJ867428
*S.dracaenae*	CBS 141323^T^	KX228283	KX228334	–	KX228377
*S.echinulatum*	GUCC 18247^T^	MZ503733	MZ503766	MZ546912	MZ546879
*S.echinulatum*	GUCC 18248	MZ503734	MZ503767	MZ546913	MZ546880
*S.ellipsoideum*	CBS 131796^T^	MN077367	–	–	–
*S.ellipsoideum*	GUCC 18264	MZ503750	MZ503783	MZ546929	MZ546896
*S.ferulica*	IRAN3232C^T^	MF186874	MH400207	–	–
*S.gamsii*	CBS 239.78^T^	KF156019	KF156150	KF156190	KF155982
*S.globale*	CBS 119644^T^	KF961086	KF961097	KF961065	KF961075
*S.globale*	CBS 135924	KF961092	KF961104	KF961070	KF961079
*S.guangxiensis*	SS23^T^	MK934570	MK956169	–	–
*S.guangxiensis*	X22	MK961215	MK961247	–	–
*S.helicteris*	NFCCI 4310^T^	MK014833	–	MK321318	–
*S.humicola*	CBS 116655^T^	HQ667521	KF156124	KF156195	KF155984
*S.icarus*	CBS 116645	HQ667525	–	LM644604	‒
*S.icarus*	CBS 536.69^T^	HQ667524	KF156132	KF156174	KF156009
*S.lascauxense*	CBS 131815^T^	FR832474	KF156136	KF156183	KF155994
*S.lascauxense*	CBS 423.64	HQ667523	KF156131	KF156173	KF156008
*S.leishanicola*	GUCC 18259	MZ503745	MZ503778	MZ546924	MZ546891
*S.leishanicola*	HGUP1808^T^	MK377301	MK377073	–	–
*S.longiphorum*	CBS 435.76^T^	KF156038	KF156135	KF156182	KF155978
*S.macrozamiae*	CBS 137971^T^	KJ869123	KJ869180	–	–
*S.minimum*	CBS 510.71^T^	HQ667522	KF156134	KF156172	KF156007
*S.mirabilis*	CBS 413.51^T^	HQ667536	KF156140	KF156164	KF156001
*S.musae*	CBS 729.95^T^	KF156029	KF156144	KF156171	KF155999
*S.musicola*	CBS 144441^T^	MH327824	MH327860	MH327898	MH327887
*S.musicola*	CPC 37308	MW063428	–	MW071116	MW071096
*S.musicola*	CPC 37309	MW063429	–	MW071117	MW071097
*S.obovoideum*	GUCC 18246^T^	MZ503732	MZ503765	MZ546911	MZ546878
*S.olivaceum*	CBS 137170^T^	LM644521	LM644564	LM644605	KT272067
*S.pandanicola*	CBS 140660^T^	KT950850	KT950864	–	–
*S.phaeophorum*	CBS 206.96^T^	KP798631	KP798634	KT272062	KT272098
*S.podocarpi*	CBS 143174^T^	MG386032	MG386085	–	–
*S.podocarpicola*	CBS 146057^T^	MN562138	MN567645	–	–
*S.ramosum*	CBS 137171	LM644522	LM644565	LM644606	KT272068
*S.ramosum*	CBS 137173^T^	LM644524	LM644567	MZ546928	KT272069
*S.robustum*	CBS 112.97^T^	KP798633	KP798636	KT272060	KT272071
*S.sexuale*	CBS 131965	KF156017	KF156119	KF156188	KF155977
*S.sexuale*	CBS 135765^T^	KF156018	KF156118	KF156189	KF155976
*S.spiralihyphum*	GUCC 18245^T^	MZ503731	MZ503764	MZ546910	MZ546877
*S.terrestre*	CBS 211.53^T^	NR_145365	NG_058014	KF156187	KF156005
*S.terreum*	CBS 203.27^T^	HQ667544	–	HQ877665	–
*S.tshawytschae*	CBS 100438^T^	HQ667562	KF156126	KF156180	KF155990
*S.tshawytschae*	CBS 228.66	KF156016	KF156128	KF156179	KF155992
*S.variabile*	NBRC 32268	DQ307334	EU107310	–	DQ307356
*S.verrucaria*	GUCC 18240^T^	MZ503726	MZ503759	MZ546905	MZ546872
*S.verrucosum*	CBS 383.81^T^	KF156015	KF156129	KF156185	KT272099
*S.zunyiense*	GUCC 18241^T^	MZ503727	MZ503760	MZ546906	MZ546873
*V.calidifluminalis*	CBS 125818^T^	AB385698	KF156108	KF156202	KF155959

**Notes**: ^a^T refers to ex-type strains. ^b^Bold indicates the sequences generated in this study. –, not applicable. **CBS**: Westerdijk Fungal Biodiversity Institute, Utrecht, the Netherlands; **CPC**: Culture collection of Pedro Crous, housed at Westerdijk Fungal Biodiversity Institute; **CGMCC**: Chinese General Microbiological Culture Collection Center, Beijing, China; **LC**: personal culture collection of Lei Cai housed in the Institute of Microbiology, Chinese Academy of Sciences; **GUCC**: Culture Collection of the Department of Plant Pathology, Agriculture College, Guizhou University, China; **HGUP**: Herbarium of the Department of Plant Pathology, Agricultural College, Guizhou University, China; **IRAN**: Fungal Culture Collections of the Iranian Research Institute of Plant Protection; **MFLU (CC)**: Mae Fah Luang University Culture Collection, Chiang Ria, Thailand; **NFCCI**: National Fungal Culture Collection of India, Pune, India; **NBRC**: Biological Resource Center.

## ﻿Results

### ﻿Phylogeny

**Figure 1.**
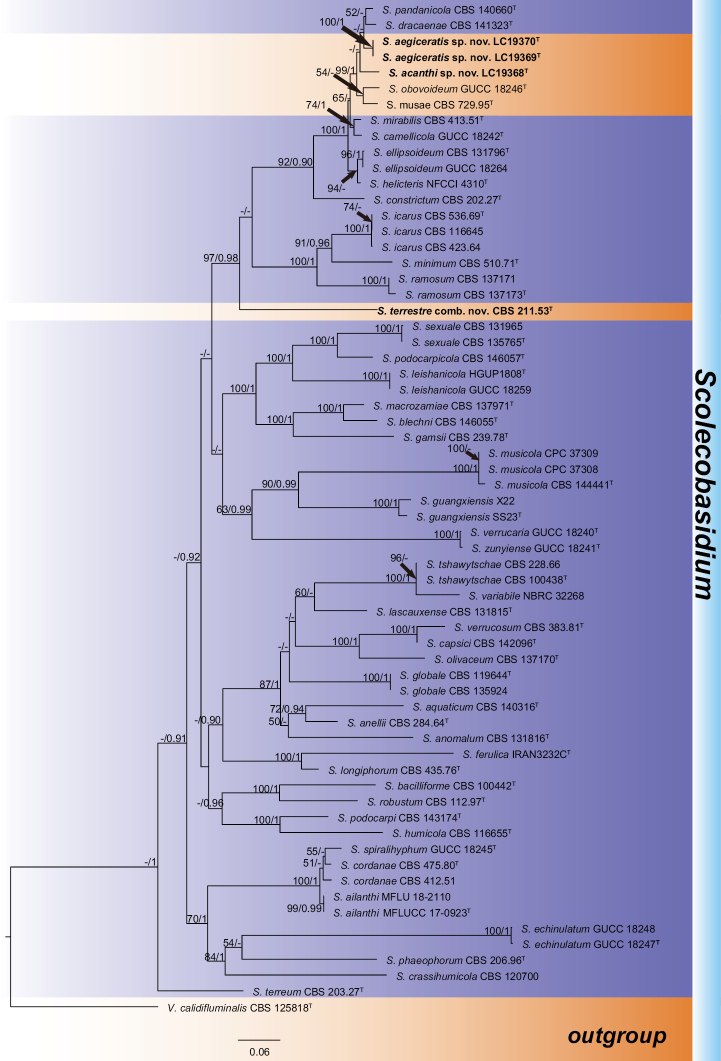
Phylogenetic tree of *Scolecobasidium* calculated with a maximum likelihood analysis of the combined ITS, LSU, *tef1*-α, and *tub2* sequences alignment. The tree was rooted with *Verruconiscalidifluminalis* (CBS 125818). Bootstrap values (ML > 50%) and Bayesian posterior probabilities (PP > 0.90) are shown at the nodes in the order of ML/PP. ^T^ indicates ex-type strains. The novel taxa and new combination are showed in bold. The scale bar represents the expected changes per site.

The BLAST searches in the NCBI’s GenBank nucleotide database using ITS sequences of LC19368, LC19369 and LC19370 showed their closest similarities to *Scolecobasidium* spp. In the following multi-locus phylogenetic analysis of *Scolecobasidium*, the dataset comprised 2,932 characters including alignment gaps (LSU: 855 bp, ITS: 818 bp, *tub2*: 542 bp, *tef1*-α: 717 bp). The ML search revealed a best tree with an InL of -34731.383727. For the Bayesian inference, a GTR+I+G model was selected for ITS, LSU, *tef1*-α and *tub2*. The BA was run for 1,535,000 generations, and a 50% consensus tree and posterior probabilities were calculated from 2,304 trees from two runs. The topologies of phylogenetic trees generated by ML and BA were congruent. Our strains were separated into two distinct clades from all known species of *Scolecobasidium* (Fig. 1).

### ﻿Taxonomy

#### ﻿ Scolecobasidium

Taxon classificationFungiVenturialesSympoventuriaceae

E.V. Abbott, Mycologia 19: 30. 1927. emend. F. Liu

E7B9FF39-4FE1-527A-A991-349D18FBB3D2

Ochroconis de Hoog & Arx, Kavaka 1: 57. 1974 [1973]. Synonym.

##### Description.

***Colonies*** restricted, slow-growing, brown or olivaceous. ***Aerial hyphae*** smooth- or somewhat rough-walled, pigmented. ***Cleistothecia*** up to 40 μm in diam, dark brown; peridium wall composed of textura angularis. ***Ascomata*** bearing antler-shaped appendages, with serrate edges. ***Asci*** bitunicate, clavate, 8-spored; ascospores pale brown, verruculose, 1–3-septate. ***Conidiophores*** reduced, unbranched or sparingly branched, arising from the aerial hyphae or hyphal ropes, continuous or septate, hyaline or pigmented, ovoid, clavate, wedge-shaped, cylindrical, or irregular. ***Conidiogenous cells*** scattered, monoblastic or sympodial, elongate to cylindrical. ***Conidia*** produced in clusters or acropetal series from the ends of tubular extensions of the conidiophores; conidia 1–4-celled, pigmented or hyaline, smooth or verrucose, ellipsoidal, ovoid, cylindrical, or T- or Y-shaped. (emended from Abbott 1927; Barron and Busch 1962; Seifert and Gams 2011; Samerpitak et al. 2014).

##### Notes.

Abbott (1927) summarized the asexual features of *Scolecobasidium* as its generic character based on only two species *S.terreum* and *S.constrictum*. Over time multiple species of *Scolecobasidium* have been described, and the boundaries between this genus and closely related genera have also been clarified (Barron and Busch 1962; Seifert and Gams 2011; Samerpitak et al. 2014; Shen et al. 2020; Wei et al. 2022). However, no one has updated the generic character of *Scolecobasidium*. In this study, we update the generic character of *Scolecobasidium* based on previous descriptions, especially the morphological features of the sexual stage of the fungus.

##### Type species.

*Scolecobasidiumterreum* E.V. Abbott.

#### ﻿ Scolecobasidium acanthi

Taxon classificationFungiVenturialesSympoventuriaceae

S. Song, L. Cai & F. Liu sp. nov.

BC4274EB-BBAD-55F3-83A5-AE92404F4E6E

847639

Fig. 2

##### Etymology.

Named after the host plant *Acanthus* from which this fungus was isolated.

##### Type.

China. Guangdong Province: Qi’ao-Dangan Island Provincial Nature Reserve, from leaf of *Acanthusebracteatus*, Nov 2019, M. Li, Z.F. Zhang and J.E. Huang (Holotype HMAS 352373, culture ex-type CGMCC 3.24352 = LC19368).

##### Description.

***Sexual morph***: unknown. ***Asexual morph***: Mycelium consisting of branched, septate, hyaline to pale brown, smooth, and thick-walled hyphae. ***Conidiophores*** solitary, erect, brown, smooth, arising from superficial hyphae, subcylindrical, straight to geniculous, brown, thick-walled, 0(–2)-septate, 14.5–20.5 × 1.5–2 µm, often reduced to conidiogenous cells, bearing a few conidia near the apex. ***Conidiogenous cells*** brown, smooth, 4.5–9.5 × 1.5–2 µm, terminal and lateral on conidiophores, containing several apical, cylindrical denticles. ***Conidia*** 1-septate, smooth-walled, subhyaline to pale brown, cylindrical, rarely pyriform, constricted at the septum, 5.5–8.5 × 2.5–4 µm (av. ± SD = 6.9 ± 0.7 × 3.05 ± 0.2 µm, n = 42).

##### Culture characteristics.

Colonies reaching up to 16–20 mm diam after 14 days at 25 °C, producing dense aerial mycelium on MEA and OA. On MEA, surface wheat to greyish brown, reverse saddlebrown, felty, dry, margins smooth. On OA, surface burlywood to peru, reverse brown black, margins smooth.

**Figure 2.**
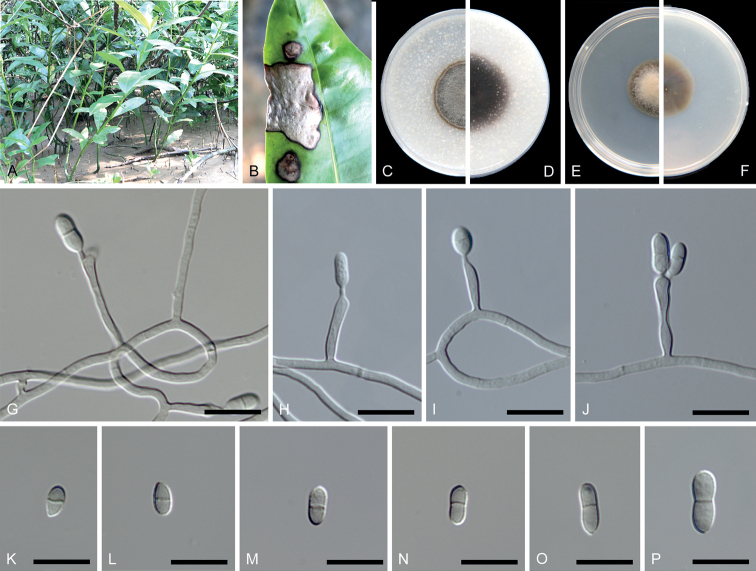
*Scolecobasidiumacanthi* (ex-type CGMCC 3.24352) **A** the habitat of *Acanthusebracteatus***B** leaf spot on *Acanthusebracteatus***C, D** forward and reverse colony on OA after 14 days **E, F** forward and reverse colony on MEA after 14 days **G–J** conidiophores, conidiogenous cells and conidia **K–P** conidia. Scale bars: 10 μm (**G–P**).

##### Notes.

Although represented by single strain, *S.acanthi* sp. nov. formed a distinct clade (Fig. 1) that was phylogenetically related to *S.aegiceratis* sp. nov. The two species differ from each other in 820/825 bp (99.39%) in LSU, 474/514 bp (92.22%) in ITS, 525/538 bp (97.58%) in *tef1*-α, and 433/464 bp (93.32%) in *tub2*. Morphologically, *S.acanthi* sp. nov. differs from *S.aegiceratis* sp. nov. in the septa number of conidiophores (0–2 vs. 0–1) and the size of conidiogenous cells (4.5–9.5 × 1.5–2 µm vs. 7.5–24 × 1.5–2.5 µm) and conidia (5.5–8.5 × 2.5–4 µm vs. 8–15(–26.5) × 2.5–3.5(–6.5) µm).

#### ﻿ Scolecobasidium aegiceratis

Taxon classificationFungiVenturialesSympoventuriaceae

S. Song, L. Cai & F. Liu sp. nov.

A69B97A0-0434-501C-931A-B1ACE1C88CD0

847640

Fig. 3

##### Etymology.

Named after the host plant *Aegiceras* from which this fungus was isolated.

##### Type.

China. Guangdong Province: Futian Mangrove National Nature Reserve, from leaf of *Aegicerascorniculatum*, July 2020, Z.F. Zhang (Holotype HMAS 352374, culture ex-type CGMCC 3.24353 = LC19369).

##### Other material examined.

China. Guangdong Province: Futian Mangrove National Nature Reserve, from leaf of *Aegicerascorniculatum*, July 2020, Z.F. Zhang (Holotype HMAS 352375, culture ex-type CGMCC 3.24354 = LC19370).

##### Description.

***Sexual morph***: unknown. ***Asexual morph***: Mycelium consisting of branched, septate, hyaline to pale brown, smooth, and thick-walled hyphae. ***Conidiophores*** arising from the aerial hyphae or hyphal ropes, continuous or septate, usually reduced to conidiogenous cells, 0(–1)-septate. ***Conidiogenous cells*** solitary, hyaline to pale brown, smooth, subcylindrical, straight to geniculous-sinuous, thick-walled, 7.5–24 × 1.5–2.5 µm, bearing a few conidia near the apex. ***Conidia*** smooth-walled, subhyaline to pale brown, ellipsoidal or cylindrical, tapering torwards the base, mostly 1-septate, rarely 2–3-septate, sometimes constricted at the septum, 8–15(–26.5) × 2.5–3.5(–6.5) µm (av. ± SD = 9.3 ± 1.16 × 2.83 ± 0.26 µm, n = 40).

**Figure 3.**
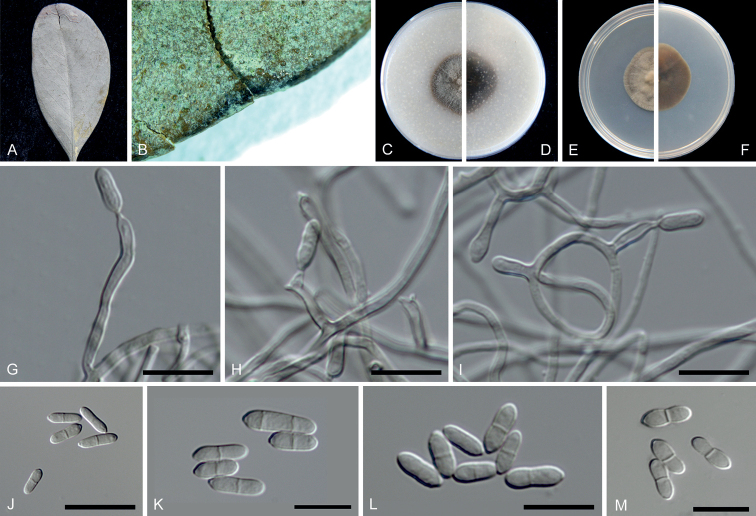
*Scolecobasidiumaegiceratis* (ex-type CGMCC 3.24353) **A, B** leaf spots of *Aegicerascorniculatum***C, D** forward and reverse colony on OA after 14 days **E, F** forward and reverse colony on MEA after 14 days **G–I** conidiophores, conidiogenous cells and conidia **J–M** conidia. Scale bars: 10 μm (**G–M**).

##### Culture characteristics.

Colonies reaching up to 20–22 mm diam after 14 days at 25 °C, dense aerial mycelium on MEA and OA. On MEA, smooth to felty, dry, surface greyish brown to dark brown, reverse saddle brown. On OA, surface ivory to peru, reverse brown black.

##### Notes.

*Scolecobasidiumaegiceratis* is phylogenetically related to *S.dracaenae* (Fig. 1) and can be differentiated from the later by DNA sequences of LSU (99.52% similarity), ITS (93.55%) and *tef1*-α (96.60%) regions. Morphological characters of the two species are overlapping but their conidiophores and conidia show differences. *Scolecobasidiumaegiceratis* can be distinguished from *S.dracaenae* as it produces hyaline to pale brown (vs. brown in *S.dracaenae*) conidiogenous cells. In addition, the dimensions of their conidia (8–15(–26.5) × 2.5–3.5(–6.5) µm vs. 6.5–10 × 3–4 μm) and conidiogenous cells (7.5–24 × 1.5–2.5 µm vs. 5–15 × 2.5–3 μm) are different (Crous et al. 2016).

#### ﻿ Scolecobasidium constrictum

Taxon classificationFungiVenturialesSympoventuriaceae

E.V. Abbott, Mycologia 19(1): 30. 1927.

C1027CD3-7A3C-5777-BF86-992C10008E5D

≡ Ochroconisconstricta (E.V. Abbott) de Hoog & Arx, Kavaka 1: 57. 1974.
≡ Dactylariaconstricta (E.V. Abbott) D.M. Dixon & Salkin, J. Clin. Microbiol. 24: 13. 1986.

##### Type.

USA Louisiana, from soil, 1927, E.V. Abbott ex-type culture CBS 202.27 = MUCL 9471 (metabolically inactive).

##### Notes.

*Scolecobasidiumconstrictum* was introduced at the same time as the generic type of *Scolecobasidium*, *S.terreum*, by Abbott (1927). Later, *Heterosporiumterrestre* was treated as a synonym of *S.constrictum* due to their similar morphological characteristics (Barron and Busch 1962). Their original descriptions differ, however, in that *H.terrestre* produces rough conidia and variable conidiophores both in shape and size, and has occasional phragmospores. Therefore, Barron (1962) thought that Abbott described only a facet of *S.constrictum* and emended the species description. Subsequently, *Ochroconis* was introduced to accommodate species with sympodial conidiogenesis and unbranched, subspherical to cylindrical or clavate, melanised conidia, and *S.constrictum* was transferred to this genus as *O.constricta* and designated as the generic type (De Hoog 1973).

With the help of molecular analyses, Shen et al. (2020) synonymized *Ochroconis* under *Scolecobasidium*, and *S.constrictum* should be resurrected as a result. We observed that the ex-type cultures of *H.terrestre* (CBS 211.53) and *S.constrictum* (CBS 202.27) were separated into two distinct clades with relatively long branches in the current study (Fig. 1) and sequences similarities between the two species were very low (LSU: 98.7%; ITS: 88%, *tef1*-α: 85%, *tub2*: 79%). Since *S.constrictum* (CBS 202.27) is now sterile (Shen et al. 2020), we could only make a detailed morphological comparison among multiple descriptions of ex-type cultures of *H.terrestre* (CBS 211.53) and *S.constrictum* (CBS 202.27) (Abbott 1927; Atkinson 1952; Barron and Busch 1962; Samerpitak et al. 2014), and found that the two fungi were morphologically different in the shape (oval to ovate, short-cylindrical and long cylindrical to sole-shaped in *H.terrestre* vs. echinulate to verrucose in *S.constrictum*) and number of septa (0–3 vs. 0–1) in the conidia, as well as the size of conidiophores (2–31.5 × 1.5–2.5 μm vs. 5–8 × 2–2.5 μm). Based on morphology combined with the phylogeny, we consider that *H.terrestre* R.G. Atk. should not be treated as a synonym of *S.constrictum*, and a new combination *Scolecobasidiumterrestre* comb. nov. is proposed in this study.

In addition, the type strain of *S.constrictum* should be CBS 202.27, rather than CBS 211.53, which was incorrectly listed in tables 2, 3 and figs 1, 2 in Wei et al. (2022).

#### ﻿ Scolecobasidium terrestre

Taxon classificationFungiVenturialesSympoventuriaceae

(R.G. Atk.) F. Liu, S. Song & L. Cai comb. nov.

302177B4-DDB6-5208-A60B-B07E12205A01

847635

≡ Heterosporiumterrestre R.G. Atk., Mycologia 44: 813. 1952.

##### Type.

Canada. Ontario: Ancaster, obtained from soil obtained, isolated by R.G. Atkinson, 31 Oct. 1947, holotype DAOM 28282, ex-type culture CBS 211.53 (= ATCC 11419; DAOM 28282; IMI 051380; MUCL 9896).

##### Note.

See the notes under *S.constrictum*.

## Supplementary Material

XML Treatment for Scolecobasidium

XML Treatment for Scolecobasidium acanthi

XML Treatment for Scolecobasidium aegiceratis

XML Treatment for Scolecobasidium constrictum

XML Treatment for Scolecobasidium terrestre
